# Multiple Myeloma in a Patient with ANKRD26-Related Thrombocytopenia Successfully Treated with Combination Therapy and Autologous Stem Cell Transplant

**DOI:** 10.1155/2019/9357572

**Published:** 2019-06-02

**Authors:** Muhammad Husnain, Trent Wang, Maikel Valdes, James Hoffman, Lazaros Lekakis

**Affiliations:** ^1^Division of Hematology/Oncology, Department of Medicine, Miller School of Medicine, University of Miami, Miami, FL, USA; ^2^Sylvester Comprehensive Cancer Center, University of Miami, Miami, FL, USA

## Abstract

Ankyrin repeat domain-containing protein 26- (ANKRD26-) related thrombocytopenia is a rare, autosomal dominant condition caused by ANKRD26 gene mutation. ANKRD26-related thrombocytopenia is characterized by moderate thrombocytopenia with minimal bleeding, normal platelet size, and dysmegakaryopoiesis on bone marrow evaluation. ANKRD26 mutation has been previously associated with myeloid malignancies, including acute myeloid leukemia, myelodysplastic syndrome, and chronic myeloid leukemia. We report the first case of multiple myeloma in a patient with ANKRD26 related thrombocytopenia. The patient was successfully treated with contemporary combination therapy followed by melphalan-conditioned autologous stem cell transplant for his multiple myeloma despite preexisting thrombocytopenia.

## 1. Introduction

Ankyrin repeat domain-containing protein 26- (ANKRD26-) related thrombocytopenia is a rare, autosomal dominant condition caused by ANKRD26 gene mutation [1]. Its clinical hallmark includes moderate thrombocytopenia with minimal bleeding, normal platelet size, and dysmegakaryopoiesis on bone marrow evaluation. Mild polycythemia and leukocytosis have also been reported [2]. There is reported association between ANKRD26 mutation with myeloid malignancies, including acute myeloid leukemia, myelodysplastic syndrome, and chronic myeloid leukemia [2]. No known correlation with multiple myeloma has been previously identified. We report a case of multiple myeloma and ANKRD26-related thrombocytopenia (ANKRD26-RT) successfully treated with combination therapy and autologous stem cell transplant.

## 2. Case Presentation

A 64-year-old Caucasian man with a lifelong history of thrombocytopenia was found with elevated total protein of 10.3 g/dl, serum monoclonal spike of 3.5 g/dl, immunoglobulin G (IgG) of 5371 mg/dl, and free lambda light chains of 703 mg/dl with free kappa/lambda ratio of 0.08. Calcium and renal function were within normal limits. Hemoglobin was 16.6 g/dL, and platelet count was 33 × 10^3^/*μ*L. Skeletal survey was negative for any lytic lesions. Bone marrow examination showed adequate megakaryocytes with normal platelet morphology, and no dysmegakaryopoiesis or micromegakaryocytes were seen on the bone marrow. Erythrocytes and granulocytes were adequate in number without any significant abnormality.

The past medical history included essential hypertension and hypogonadism, but without neuropathy or dermatologic conditions. The diagnosis of thrombocytopenia stemmed from childhood and was accompanied by a penetrant family history of thrombocytopenia seen in his mother, maternal aunt, brother, and son. No genetic cause of the thrombocytopenia had been established, and questioning found no personal or family history of physical deformity, vision, hearing, or bleeding disorders. Laboratory review of his platelet count history ranged from 30 to 50 × 10^3^/*μ*L.

Repeat testing of hemoglobin was 17.9 g/dl with hematocrit of 55.6%. Peripheral blood examination showed normal appearing red and white blood cells, with erythrocytosis, and morphologically normal platelets that were decreased in number. Erythropoietin level was normal (12.2 mIU/mL), and molecular testing found no mutations in JAK2 V617F or BCR/ABL1 (p190 and p210). The erythrocytosis was attributed to use of testosterone supplementation for hypogonadism. A limited congenital thrombocytopenia testing panel was ordered and resulted negative for mutations in myeloproliferative leukemia protein (MPL), runt-related transcription factor 1 (RUNX1), myosin heavy chain 9 (MYH9), and Wiskott–Aldrich syndrome (WAS) gene mutations. Flow cytometry for platelet associated autoantibodies. Anti-glycoprotein IIb/IIIa antibodies, anti-glycoprotein Ib/Ix antibodies, and anti-glycoprotein CD36 antibodies were also negative.

After a period of monitoring, his serum IgG increased to 7063 mg/dl and monoclonal-spike to 5.4 g/dl. Albumin level was 2.7 g/dl, and beta-2 microglobulin level was 2.52 mg/dl. Repeat bone marrow biopsy showed 70% plasma cells with adequate megakaryocytes and normal platelet morphology, and he was diagnosed with International Staging System (ISS) stage II IgG lambda multiple myeloma. Treatment was now indicated, however complicated given his marked thrombocytopenia.

He started treatment with pulse doses of dexamethasone (40 mg daily for 4 days) with subsequent addition of bortezomib at 0.4 mg/m^2^ with slow escalation to full dose (1.3 mg/m^2^) by week 3. Oral cyclophosphamide (600 mg/weekly) was added to this regimen during cycle 2. After 5 cycles of CyBorD (cyclophosphamide, bortezomib, and dexamethasone), the patient had normalization of IgG levels and decrease in M-spike to 0.62 g/dl consistent with a partial response. He was referred for consolidative melphalan-conditioned autologous stem cell transplant.

Stem cell mobilization with granulocyte colony-stimulating factor (10 mcg/kg × 5 doses and plerixafor 24 mg × 1 dose) resulted in peak white blood cell count of 151 × 10^3^/*μ*L and stem cell yield of 105.6 × 10^6^ CD34+/kg in a single session (the highest recorded at our institution that year). The patient proceeded to high-dose melphalan (200 mg/m^2^) followed by infusion of 45 × 10^6^ CD34+/kg autologous stem cells. Despite a robust and quick neutrophil engraftment on day +9, the patient encountered marked nausea and a severe pruritic skin rash that started after engraftment and lasted for 1-2 months, resembling a graft versus host reaction. After engraftment, his platelet count peaked as high as 84 × 10^3^/*μ*L on *D* + 21 before slowly declining back to his baseline of about 30 × 10^3^/cells 6 weeks after transplant. He continued to have stable disease after autologous transplant and started lenalidomide maintenance with aspirin prophylaxis.

At a subsequent follow-up visit after autologous transplant, a more comprehensive 21-gene-inherited thrombocytopenia panel was sent, and a heterogeneous mutation of ANKRD26 c.-128G>A in the 5′ untranslated region (5′ UTR) of chromosome 10 was identified. This was consistent with a diagnosis of ANKRD26-RT. The patient continues to have persistent thrombocytopenia in the range of 30–50 × 10^3^ platelets. Given his significant family penetrance of thrombocytopenia, the patient and family were referred to genetic counseling for further evaluation.

## 3. Discussion

Point mutations in the 5′ UTR of ANKRD26 have now been reported in almost 60 unrelated families accounting for almost 200 cases of ANKRD26-RT [2, 3]. There are total of 17 different heterozygous mutations identified to date in the 5′ UTR of ANKRD26 with nucleotide substitutions at position c.-118C>T, c.-128G>T, and c.134G>A representing 75% of all mutant alleles [2, 3].

The mechanism by which ANKRD26 promotes thrombocytopenia is not clearly understood but the MAPK/ERK1/2 pathway has been implicated. ANKRD26 overexpression in megakaryocytes increases MAPK/ERK pathway activity in megakaryocytes and alters TPO/MPL function leading to thrombocytopenia [4]. CXCR4-SDF1 axis is also implicated in pathogenesis of ANRD26-RT as it directs megakaryocyte migration towards the vascular niche and permits terminal maturation and proplatelet formation [5]. Decreased expression of CXCR4 may lead to decreased terminal maturation of platelets and thus thrombocytopenia. Noris et al. have described 78 patients of ANKRD26-related thrombocytopenia from 21 families [1]. They have reported an increased number of megakaryocytes at all stages of maturation in bone marrows of these patients with dysmegakaryopoiesis, small megakaryocytes with hypolobulated nuclei, and typical micromegakarycytes. In our case, we did not see any dysmegakaryopoiesis, and the megakaryocytes in our patient were normal in number and morphology. Mild polycythemia and leukocytosis have also been reported in a small subset of patients with congenital thrombocytopenia, however without clear explanation [1]. In our case, the patient's polycythemia may have been attributed to preexistent testosterone supplementation use.

It is known that patients with ANKRD26 mutations are at increased risk of developing hematological malignancies including myelodysplastic syndrome, acute myeloid leukemia, and chronic myeloid leukemia. In the largest series of 118 patients with ANKRD26-RT, 10 (8%) ultimately developed myeloid malignancies [2]. Loss of RUNX1 and friend leukemia integration 1 transcription factor (FLI1) and increased signaling of MPL have been implicated in the pathogenesis of myeloid malignancies in the patients with ANKRD26 mutations [4]. ASXL1-mutated chronic myelomonocytic leukemia has also been reported in a patient with ANKRD26-RT [6]. Zhang et al. have previously described hereditary association between thrombocytopenia and hematological malignancies including one case of multiple myeloma in three families with germline ETV6 mutation [7], but there is no reported case of ANKRD26 RT and multiple myeloma. To our knowledge, this is the first case of multiple myeloma in a patient with ANKRD26-RT. While it is unclear whether this relationship was an incidental finding, ANKRD26 mutation was identified in 3.1% of African-American patients and 0.2% of Caucasian patients in a molecular profiling study of 718 multiple myeloma patients [8]. It is not known whether these mutations of ANKRD26, detected with a higher frequency in African American than in Caucasian multiple myeloma patients, were preferentially localized in the 5′ UTR part of this gene.

We demonstrated safe and successful treatment of multiple myeloma using contemporary combination therapy followed by melphalan-conditioned autologous stem cell transplant despite preexisting thrombocytopenia. Of interest, mobilization with filgrastim and plerixafor resulted in marked leukocytosis and extraordinary yield of CD34+ stem cells after only one session of apheresis. We postulate this may have been related to decreased surface CXCR4 expression which has been reported in association with ANKRD26-RT [5] along with CXCR4 antagonism by plerixafor, thus amplifying CXCR4 blockade.

The appearance of an autologous graft-versus-host skin eruption and the transient rise of platelets above pretransplant baseline remain unexplained. Perhaps, a rebound effect of increased CXCR4 after stem cell mobilization and posttransplant use of granulocyte colony stimulating factor contributed to the brisk yet temporary rise in platelets.

## 4. Conclusion

This to our knowledge is the first reported case of multiple myeloma in a patient with ANKRD-26RT. We demonstrate the safe and successful treatment of multiple myeloma using contemporary combination therapy followed by melphalan-conditioned autologous stem cell transplant despite preexisting thrombocytopenia.
